# Assessing the risk of self-diagnosed malaria in urban informal settlements of Nairobi using self-reported morbidity survey

**DOI:** 10.1186/1475-2875-6-71

**Published:** 2007-05-26

**Authors:** Yazoumé Yé, Elizabeth Kimani-Murage, John Kebaso, Frederick Mugisha

**Affiliations:** 1 African Population and Health Research Centre (APHRC), Shelter Afrique Centre, 2nd Floor, Longonot Road, Upper Hill, P.O. Box 10787-00100 GPO, Nairobi, Kenya

## Abstract

**Background:**

Because of the belief that Nairobi is a low risk zone for malaria, little empirical data exists on malaria risk in the area. The aim of this study was to explore the risk of perceived malaria and some associated factors in Nairobi informal settlements using self-reported morbidity survey.

**Methods:**

The survey was conducted from May to August 2004 on 7,288 individuals in two informal settlements of Nairobi. Participants were asked to report illnesses they experienced in the past 14 days. Logistic regression was used to estimate the odds of perceived-malaria. The model included variables such as site of residence, age, ethnicity and number of reported symptoms.

**Results:**

Participants reported 165 illnesses among which malaria was the leading cause (28.1%). The risk of perceived-malaria was significantly higher in Viwandani compared to Korogocho (OR 1.61, 95%CI: 1.10–2.26). Participants in age group 25–39 years had significantly higher odds of perceived-malaria compared to those under-five years (OR 2.07, 95%CI: 1.43–2.98). The Kikuyu had reduced odds of perceived-malaria compared to other ethnic groups. Individuals with five and more symptoms had higher odds compared to those with no symptoms (OR 23.69, 95%CI: 12.98–43.23).

**Conclusion:**

Malaria was the leading cause of illness as perceived by the residents in the two informal settlements. This was rational as the number of reported symptoms was highly associated with the risk of reporting the illness. These results highlight the need for a more comprehensive assessment of malaria epidemiology in Nairobi to be able to offer evidence-based guidance to policy on malaria in Kenya and particularly in Nairobi.

## Background

Malaria remains a major public health burden in sub-Saharan Africa (SSA) [1-3]. The disease is endemic in Kenya, where it exerts a heavy burden on the health system. Malaria is the leading cause of outpatient morbidity in six provinces out of eight [4] and accounts for more than 8.2 million outpatient treatments at the government's health facilities each year. Although the disease is endemic in the country, the level of transmission varies according to the ecological settings. The country is divided into four malaria epidemiological zones namely:-endemic zone, highland (epidemic prone) zone, arid (epidemic prone) zone and low risk zone [5]

Nairobi, the capital city of Kenya, is classified as a low risk area and, therefore, malaria is not considered a major public health problem. The city is located 1,700 metres above sea level and the cold seasonal temperatures limit development of *Plasmodium falciparum *sporozoite stage in the salivary glands of the mosquito vector [6-14]. In addition, the urban habitat is less suitable for *Anopheles gambiae s.s. *and *Anophele funestus*, the dominant malaria vectors in sub-Saharan Africa [15,16]. A survey undertaken in 1982 among residents at nine sites across the city revealed a low range of *P. falciparum *infection prevalence estimates from 1.8% to 13.5% with an average of 4.9% [17]. Despite the expected low transmission, malaria continues to be a common diagnosis made among out-patient attendants to clinics in the city. For example, in 2001, malaria was ranked as the second highest contributor to outpatient morbidity accounting for 10.6% of morbidity in Nairobi after respiratory system diseases [4].

Does the population who live in informal settlements also perceive malaria as one of the key morbidities? Their perception of malaria will most likely influence their treatment-seeking behaviour and consequent use of anti-malarial drugs. This study, seeks to assess the level of perceived-malaria and the associated factors using self-reported morbidity survey in Nairobi informal settlements. Data from a self-reported morbidity survey conducted in 2004 in two informal settlements of Nairobi (Viwandani and Korogocho) was used. The survey was conducted as part of the Nairobi Urban Health and Demographic Surveillance System (NUHDSS) by the African Population and Health Research Centre (APHRC).

## Methods

### Study site

The study was part of the Nairobi Urban Health and Demographic Surveillance System (NUHDSS), which is run by APHRC. It covers two informal settlements of Korogocho and Viwandani in Nairobi city. Both are informal settlements located about 5–10 km from the city centre and 3 km from each other. The population under surveillance in 2004 was 59,698 inhabitants, with 26,533 living in Korogocho and 33,165 in Viwandani. Poor environmental sanitation, overcrowded houses and poor access to basic health care characterize these settlements [18,19]. These conditions impact dramatically on their health. Compared to the rest of Kenya, the informal settlements exhibit worse health indicators, especially for the under-five population [18,20,21]. Insecticide-treated net (ITN) possession is expected to be low in the informal settlements. In reality, according to the DHS 2003, the proportion of households in Nairobi province who possesses ITN is 6.9%, which is only slightly higher than the national level (5.9%). However, within the lowest wealth quintile only 2.5% of the household possess an ITN [22]. Since most of slum-dwellers do fall within the lowest wealth quintile, ITN coverage is expected to be low therein.

### Study population

The household survey covered 7,288 individuals randomly selected from the population under surveillance. Analysis was restricted to those individuals who reported at least one illness during the morbidity survey. The population of interest is, therefore, comprised of 1,394 individuals.

### Self-reported morbidity survey

The self-reported morbidity survey was conducted as part of a panel household survey in Korogocho and Viwandani from May to August 2004. During this survey 7,288 individuals were interviewed. They were asked to report a maximum of three illnesses they had during the past 14 days as well as the symptoms. For the symptoms, respondents were asked to report at most five symptoms. Data for individuals below 14 years old were collected from proxy respondents (parents or caretakers).

### Statistical analysis

Data were processed and cleaned in MS Access and exported to STATA 9 for statistical analysis. Logistic regression was used to estimate the odds of reporting malaria given specific factors. The multivariate model included factors such as site of residence (Viwandani compared to Korogocho), gender (male compared to female), age groups (5–14 years, 15–24 years, 25–39 years and 40+ years, compared to under-five years the most susceptible population in high transmission and endemic area [23,24], ethnicity (Kamba, Luhya and Luo compared to Kikuyu) and a symptom score (Score 1, Score 2, Score 3, Score 4 and Score 5+ compared individually to Score 0). To obtain the symptom score variable, we first selected symptoms which are likely to be associated with malaria among the 29 listed. These are (a) fever, (b) convulsions, (c) headache, (d) vomiting, (e) joint pain, (f) tiredness, (g) loss of appetite, (h) chest pain, (i) abdominal pain, (j) diarrhoea, and (k) paralysis. The score was then computed by counting the number of symptoms reported in the presence of fever. An individual would score zero if he reported none of these symptoms, score 1 if he reported only fever and score more than one to those who reported fever + another of the malaria-related symptoms. To illustrate how the score works, consider an individual A with fever only. Consider individual B with fever, vomiting and joint pains. And consider individual C with sleeping difficulty. Individuals A, B, and C get scores 1, 3, and 0 respectively. The logistic regression model was defined as follows:

log*it*(*π*_*i*_) = *β*_0 _+ *β*_1_*Slum_Vivandani*_*i *_+ *β*_2_*Gender_Male*_*i *_+ *β*_3_*AgeGroup_*15 – 24_*i *_+ *β*_4_*AgeGroup_*24 – 39_*i *_+ *β*_5_*AgeGroup_*40_*i *_+ *β*_6_*Ethnicity_Kamba*_*i *_+ *β*_7_*Ethnicity_Luha*_*i *_+ *β*_8_*Ethnicity_Luo*_*i *_+ *β*_9_*Symptom_Score_*1_*i *_+ *β*_10_*Symptom_Score_*2_*i *_+ *β*_11_*Symptom_ Score_*3_*i *_+ *β*_12_*Symptom_ Score_*4_*i *_+ *β*_13_*Symptom_ Score_*5 + _*i*_

Where *π*_*i *_is the predicted probability of reporting malaria of the *i*th individual; the odds of the same individual will be πi1−πi
 MathType@MTEF@5@5@+=feaafiart1ev1aaatCvAUfKttLearuWrP9MDH5MBPbIqV92AaeXatLxBI9gBaebbnrfifHhDYfgasaacH8akY=wiFfYdH8Gipec8Eeeu0xXdbba9frFj0=OqFfea0dXdd9vqai=hGuQ8kuc9pgc9s8qqaq=dirpe0xb9q8qiLsFr0=vr0=vr0dc8meaabaqaciaacaGaaeqabaqabeGadaaakeaadaWcaaqaaGGaciab=b8aWnaaBaaaleaacqWGPbqAaeqaaaGcbaGaeGymaeJaeyOeI0Iae8hWda3aaSbaaSqaaiabdMgaPbqabaaaaaaa@352D@. *β*_0 _is the intercept, and *β*_1 _...*β*_13 _the regression coefficients of the independent variables (name following each coefficient). The odds ratio of reporting malaria associated with *Slum_Viwandani *compared to *Slum_Korogocho *(reference) is the exponential of *β*_1 _(OR _*Slum_Viwandani *_= exp (*β*_1_)). Statistical significance was assessed using 95% confidence intervals (CI).

## Results

### Study population characteristics

Table 1 shows the study participants' characteristics. In total 1,394 individuals took part in the study. 58.7% of them were from Korogocho and 41.3% from Viwandani. There were more males (53.1%) than females (46.9%). The age distribution shows a high proportion of under-fives (24.5%) and 25–40 year age group (26%). The other age groups represented almost the same number of individuals. The median age was 21 years, range: 0 to 75 years. Participants were classified in four major ethnic groups which are Kikuyu, Kamba, Luhya and Luo. Kikuyu (26.3%) are the majority followed by Kamba (22.3%), Luo (21.9%) and Luhya (14.3%). Ten other ethnic groups were grouped in the category "Other" (15.4%).

**Table 1:** Characteristics of study populations

**Variables**	**N**	**Percent**
**Total Population**	**1394**	**100**
		
**Slums**		
Korogocho	818	58.7
Viwandani	576	41.3
		
**Gender**		
Female	654	46.9
Male	739	53.0
		
**Age group (Years)**		
Under 5	343	24.6
5–14	235	16.9
15–24	214	15.4
25–40	367	26.3
>40	235	16.9
Age median	21	(0–75)
		
**Ethnicity**		
Kikuyu	367	26.3
Kamba	311	22.3
Luha	199	14.3
Luo	305	21.9
Other	212	15.2
		
**Symptoms Scores**		
Score 0	413	29.6
Score 1	139	10.0
Score 2	291	20.9
Score 3	290	20.8
Score 4	168	12.1
Score 5+	93	6.7

### Leading reported illness

Participants reported 165 illnesses, among which the top five causes of illness were malaria (28.1%) followed by common cold (23.4%), typhoid (2.6%), pneumonia (2.5%) and asthma (2.2%). The remaining 160 reported illnesses accounted for less than 2% of illnesses. There was a significant number (11.8%) of participants who could not identify the cause of their illness (Table 2).

**Table 2:** The top fives reported illness among the study participants

**Illnesses**	**cases**	**Percent**
Malaria	392	28.1
Common cold	320	23.0
Typhoid	36	2.6
Pneumonia	35	2.5
Asthma	30	2.2
Other (160 illnesses)	416	29.8
NOT KNOWN	165	11.8

Total	1394	100

### Perceived-malaria

Overall 392 cases of malaria were reported, which represents 28.1% of the illnesses. There was a significant difference between the two settlements whereby a higher percentage of perceived-malaria was observed in Viwandani compared to Korogocho (46.9% vs. 34.1, p-value = 0.008). No significant difference was observed between males and females (26.6% vs 29.5%, p-value = 0.25). The perceived-malaria varied significantly by age group. The age group that reported the highest malaria in descending order was 25–40 years (37.1%), the 15–24 year (28.0%), 40 years plus (27.7%), under-fives (23.6%), and the 5–14 years (21.3%). The Luo ethnic group reported the highest prevalence of malaria (35.4%) followed by Kamba (32.5%), Luhya (31.7%) and Kikuyu (22.6%). The perceived-malaria increased with the increased in number of symptoms (Table 3). For example, 54.8% of individuals who scored 5+ on the symptom scale reported malaria compared to just 5.8% who scored 0. The higher the score on the symptom scale was the more they were likely to report malaria.

**Table 3:** Distributions of self-reported malaria as illness among study participants

**Variable**	**N**	**Cases**	**Prevalence**	***x*^2^*test: p*-value**
**Total Population**	1394	392	28.1	
				
**Slums**				*p *= 0.008
Korogocho	610	208	34.1	
Viwandani	392	184	46.9	
				
**Gender**				*p *= 0.25
Female	654	174	26.6	
Male	739	218	29.5	
				
**Age group (Years)**				*p *< 0.001
Under 5	343	81	23.6	
5–14	235	50	21.3	
15–24	214	60	28.0	
25–40	367	136	37.1	
>40	235	65	27.7	
				
**Ethnicity**				*p *< 0.001
Kikuyu	367	83	22.6	
Kamba	311	101	32.5	
Luha	199	63	31.7	
Luo	305	108	35.4	
Other	212	37	17.5	
				
**Symptoms Scores**				*p *< 0.001
Score 0	413	24	5.8	
Score 1	139	15	10.8	
Score 2	291	101	34.7	
Score 3	290	125	43.1	
Score 4	168	76	45.2	
Score 5+	93	51	54.8	

### Factors associated with odds of perceived-malaria

The risk of perceived-malaria was significantly associated with the settlement of residence. Individuals living in Viwandani had high odds of reporting malaria compared to those living in Korogocho (OR 1.61, 95% CI: 1.10–2.26). Gender was not associated with the risk of reporting malaria. Comparing older age groups to the under-fives we observed that only the age group 25–39 years had significantly higher odds (OR 2.07, 95%CI: 1.43–2.98) of reporting malaria. Ethnicity was highly associated with the odds of reporting malaria whereby all ethnic groups have significantly higher odds than the Kikuyu. The odds of reporting malaria increased significantly with increasing symptom score. Individuals with five and more symptoms had significantly higher odds compared to those with no symptoms (OR 23.69, 95% CI: 12.98–43.23) (Table 4).

**Table 4:** Odd ratios for self-reported malaria as illness using logistics regression

**Variables**	**Odd Ratio**	***P*-value**	**95%CI**
**Slums**			
Korogocho	1		
Viwandani	**1.61**	**0.00**	**1.10 – 2.26**
			
**Sex**			
Female	1.00		
Male	1.13	0.37	0.86 – 1.48
			
**Age group (Years)**			
Under 5	1.00		
5–14	1.03	0.90	0.66 – 1.59
15–24	1.31	0.22	0.85 – 2.02
25–39	**2.07**	**0.00**	**1.43 – 2.98**
>40	1.32	0.20	0.86 – 2.01
			
**Ethnicity**			
Kikuyu	1.00		
Kamba	**1.50**	**0.04**	**1.10 – 2.22**
Luha	**2.21**	**0.00**	**1.42 – 3.44**
Luo	**2.12**	**0.00**	**1.45 – 3.11**
			
**Symptoms Scores**			
Score 0	1.00		
Score 1	**2.13**	**0.04**	**1.05 – 4.32**
Score 2	**9.44**	**0.00**	**5.76 – 15.47**
Score 3	**13.61**	**0.00**	**8.37 – 22.13**
Score 4	**14.89**	**0.00**	**8.77 – 25.28**
Score 5+	**23.69**	**0.00**	**12.98 – 43.23**

* 95% Confidence Limit, bold = significant at 0.05% level

## Discussion

The Kenya National Malaria Strategy [25] outlines interventions aimed at communities living in rural, high transmission areas. There is no direction on alternative approaches to disease prevention and diagnosis in urban settlements of presumed low risk and particularly in the informal settlements. The results of this study demonstrated that malaria may be a significant health problem in Nairobi and particularly in the informal settlements. It is the most reported illness in the two communities. Individual health-seeking behaviour is driven by perceptions about illness and so does the choice of treatment. With self-reported malaria, one would expect a high use of anti-malarial drugs both in self-treatment [26] and at health facilities, which if wrongly treated may in turn create unnecessary drug pressure leading to resistance

Almost all the significant factors associated with perceived-malaria were associated with high levels of mobility to and from the informal settlement communities. The under fives are most susceptible to malaria yet our results suggest the 25–40 as the age bracket that reported most malaria. This age group is also the most mobile [27]. Evidence also suggests the Kikuyus (from Nairobi regions) are the least likely to be mobile to areas with endemic malaria compared to the Luo's and Luhya. The latter ethnic groups are from the western part of the country classified as the most malaria endemic region with a high-risk of transmission year around [5]. They are more likely to travel to their region for family visit often and are therefore exposed to malaria infection. As reported by Shanks and colleagues, travelling from low risk malaria area to a high-risk area is highly associated with risk of infection [28].

In addition, there is more mobility in Viwandani than in Korogocho because of the nature of the settlement [18]. Indeed Viwandani is located in the industrial area and is mainly populated by migrant workers coming to work in the factories and return to their rural homes seasonally. Therefore, there is a constant flow of the population between the other regions of the country, including those that are malaria endemic and this informal settlement. Korogocho is populated by more or less long-term dweller populations, not as mobile as their counterparts from Viwandani. This would, therefore, suggest that the malaria reported was imported or peoples' perception of malaria was based on their previous experiences in endemic areas, where fever is commonly attributed to malaria.

Despite the evidence that, the self-reported malaria is likely to be imported from endemic part of the country, the possibility that transmission is taking place locally cannot be ruled out. Indeed a significant proportion of so-called stable parts of the population have also reported malaria. Also, Nairobi is not a malaria-free zone, although the transmission may be low. Indeed, TJ Anderson reported the presence of both *Anophele. gambiae *and *Anophele funestus *in 1912 [29] and confirmed through a more detailed investigation in 1926 [30]. A school survey of infections conducted in 1929 showed that 2.5% of children were infected with *P. falciparum *[31]. The concern is even high since this study has shown a higher prevalence 5.4% (392 cases reported among 7288 participants). Compared to high-risk regions where the prevalence is above 5.4%, this might appear negligible, but looking at it critically, 5% of 60,000 inhabitants from the two informal settlements are 3,240 individuals.

The approach used to assess malaria diagnosis could be questionable, since individuals were asked to report without any laboratory test or clinical diagnosis. However, it can also be argued that only few (5.8%) reported to have malaria without fever. The majority reported malaria only if they had fever and others symptoms associated with malaria. More reported symptoms were highly associated with the risk of perceived-malaria. As it is the case in the clinic, fever was the basis of reporting malaria. It is, therefore, evident that the rational to report malaria is not that different from what is commonly used in the clinics as required by the presumptive treatment approach [5].

The evidence from this study suggests that malaria does exist in Nairobi and particularly in the informal settlements. Whether this is an element of misdiagnosis or imported malaria from other endemic areas should be a concern for those in policy and programmes. The consequence of not recognizing the malaria problem in Nairobi when it exists will increase the likelihood of misdiagnosis and complications resulting from either delayed treatment or improper treatment. On the other hand, continuing to diagnose malaria wrongly in clinics increases pressure on the anti-malarial drugs being used. It is important to wait for malariametric surveys as an alternative source of evidence. However it is critical for those in policy and programmes to begin to look at how malaria is diagnosed  and treated, keeping in mind the likelihood that malaria prevalence in Nairobi is comparable to other endemic areas.

Self-reported malaria is the most prevalent cause of morbidity in Nairobi's informal settlements. With the underlying belief that there is little malaria in Nairobi province in Kenya, no priority is given to address the problem of malaria in the slums. In Africa, malaria epidemiological research has traditionally focused on areas of stable, endemic transmission where infection with the parasite is frequent. In these areas functional clinical immunity is acquired early in life and the overall risks of morbidity and mortality from infection with *P. falciparum *are high. Conversely very little is known about the infection and clinical epidemiology of malaria in settings with low transmission, which are ecologically unsuitable for mosquito bleeding and with malaria imported elsewhere such as the urban slum communities. There is a need to verify the reported cases of malaria with more detailed community based malariametric surveys (or at least by confirming suspected cases by using microscopy or rapid diagnostic tests) to provide guidance to policy on malaria in the Nairobi.

## Competing interests

The author(s) declare that they have no competing interests.

## Authors' contributions

YY conceptualized the paper, did the analysis and wrote the manuscript.

EK-M substantially participated in the conception and writing-up of the manuscript.

JK substantially participated in the conception and writing-up of the manuscript.

FM substantially participated in the conception, analysis and writing-up of the manuscript.
